# Transcriptomic Profiling Identifies Potential Prognostic Genes in Vietnamese Patients with Non-Small-Cell Lung Cancer

**DOI:** 10.3390/cimb48050491

**Published:** 2026-05-09

**Authors:** Tuan Quoc Bach, Giang Thi Chau Truong, Bang Ngoc Dao, Thang Ba Ta, Thuy Thi Bich Vo

**Affiliations:** 1 Military Hospital 103, Vietnam Military Medical University, 261 Phung Hung, Ha Dong, Hanoi 100000, Vietnam; 2 Institute of Biology, Vietnam Academy of Science and Technology, 18 Hoang Quoc Viet, Nghia Do, Hanoi 100000, Vietnam

**Keywords:** non–small cell lung cancer, transcriptome sequencing, protein–protein interaction network, module analysis, centrality analysis, survival analysis

## Abstract

Background/Objectives: Non-small-cell lung cancer (NSCLC) is one of the most common malignancies in Vietnam, yet its molecular mechanisms remain incompletely understood. This study aimed to identify prognostic genes in Vietnamese NSCLC patients using integrative transcriptomic and bioinformatics analyses. Methods: RNA-seq data from 30 Vietnamese NSCLC patients treated at Military Hospital 103 (January 2023–April 2024) were analyzed and cross-validated with the Gene Expression Omnibus (GEO) dataset GSE140343 to identify shared differentially expressed genes (DEGs). Subsequent analyses included functional enrichment (GO and KEGG), protein–protein interaction (PPI) network construction via STRING, and module/centrality analyses to pinpoint hub genes. Finally, prognostic significance was evaluated using overall survival data from The Cancer Genome Atlas (TCGA) via the GEPIA platform. Results: A total of 1900 shared DEGs were identified, most of which were enriched in cancer-related pathways. The resulting PPI network (comprising 1528 nodes and 8185 edges) yielded eight significant modules containing 64 high-centrality candidate genes. Survival analyses demonstrated that high expression of *CCNA2* and *S100A12*, and low expression of *ADRB2*, *ARRB1*, *PTGS2*, and *SMAD7* were significantly associated with poor overall survival in NSCLC patients. Conclusions: These findings highlight potential biomarkers for prognosis and may inform future therapeutic strategies in Vietnamese NSCLC patients.

## 1. Introduction

Lung cancer remains the leading cause of cancer-related mortality worldwide. According to the World Health Organization and the International Agency for Research on Cancer, more than 2.2 million new lung cancer cases and approximately 1.8 million deaths were reported globally in 2022, accounting for nearly one in five cancer-related deaths [1]. In Vietnam, lung cancer is the second most common cause of cancer mortality, with over 22,000 deaths annually, highlighting a substantial public health burden [2]. NSCLC represents approximately 85% of all lung cancer cases and comprises several histological subtypes, primarily lung adenocarcinoma (LUAD) and lung squamous cell carcinoma (LUSC) [3]. Despite major advances in early detection and therapeutic strategies, including surgery, chemotherapy, targeted therapy, and immunotherapy, the overall 5-year survival rate for NSCLC remains low, particularly for patients diagnosed at advanced stages [4,5]. Clinical heterogeneity among patients with similar tumor stages suggests that molecular and genetic factors play a crucial role in disease progression and therapeutic response [6].

High-throughput transcriptomic technologies, particularly RNA sequencing (RNA-seq), have enabled comprehensive profiling of gene expression changes associated with tumorigenesis. Combined with bioinformatics approaches such as differential expression analysis, PPI network construction, module detection, and network centrality analysis, transcriptomic data can reveal key regulatory genes and signaling pathways involved in cancer progression [7,8,9]. These system-level approaches are increasingly used to identify novel biomarkers for cancer diagnosis, prognosis, and therapeutic targeting.

However, most large-scale transcriptomic studies of NSCLC have been conducted using publicly available datasets such as TCGA and the GEO, which predominantly represent Western populations [10]. Transcriptional landscapes may vary across populations due to differences in genetic background, environmental exposure, lifestyle factors, and tumor biology [11]. Consequently, the applicability of biomarkers identified in Western cohorts to Southeast Asian populations remains uncertain.

To address this gap, we analyzed RNA-seq data from 30 Vietnamese patients with NSCLC and integrated these data with the GEO dataset GSE140343 to identify robust shared differentially expressed genes. Using functional enrichment, PPI network construction, module analysis, and centrality-based network approaches, we identified potential hub genes and evaluated their prognostic relevance using independent TCGA survival data. This study aims to provide population-relevant molecular insights into NSCLC and to identify candidate prognostic biomarkers for future validation.

## 2. Materials and Methods

### 2.1. Data Preparation

Total RNA was isolated from biopsy tissue of an NSCLC tumor using TRIzol reagent (Thermo Fisher, Waltham, MA, USA) according to the manufacturer’s instructions. RNA degradation and contamination were assessed by electrophoresis on 1% agarose gels, and RNA purity was evaluated using a NanoPhotometer spectrophotometer (Implen GmbH, Munich, Germany). For each sample, 1 μL of total RNA was used as input material for complementary DNA (cDNA) synthesis using the Tetro™ cDNA Synthesis Kit (Bioline, Meridian Bioscience, London, UK). The resulting cDNA libraries were subsequently subjected to high-throughput sequencing to generate transcriptome profiles.

Raw sequencing data were subjected to quality control to remove low-quality reads and potential technical artifacts. Clean reads were then aligned to the reference genome GRCh38.p14 before being quantified by featureCounts (version 1.6.3) [12]. DEGs were identified by DESeq2 package (version 1.48.1) in the R software (version 4.5).

To enhance the robustness of DEGs identification and minimize false-positive results associated with the limited sample size, an independent RNA-seq dataset from the GEO; GSE140343 [13] was incorporated for cross-validation. DEGs within the GEO dataset was identified by GEO2R (https://www.ncbi.nlm.nih.gov/geo/geo2r/, accessed on 9 April 2026), an online tool in the GEOquery (version 2.76.0) and limma packages (version 3.66.0) [14]. The adjusted *p*-value (*p*_adj_) was regarded as the standard to correct the occurrence of false-positive results using the Benjamini–Hochberg false discovery rate method. The cut-off conditions were set to the absolute value of log-fold change greater than 1 and an adjusted *p*-value less than 0.01, which was defined as statistically significant for the DEGs. The genes whose value of log-fold change was positive were marked as highly expressed genes, whereas those whose value of log-fold change was negative were marked as low expressed genes. Only genes consistently identified as differentially expressed in both datasets were considered robust DEGs, were illustrated by Venny 2.1.0 [15], and were selected for subsequent protein–protein interaction network construction and further analyses.

### 2.2. Functional Enrichment Analyses

GO and KEGG are used to understand and simulate higher-order functional behaviors of cells or organisms from the genomic information. GO and KEGG annotations of shared DEGs were carried out through the DAVID database (https://david.ncifcrf.gov/, accessed on 9 April 2026), an online program providing a comprehensive set of functional annotation tools [16,17]. The analysis of Gene Ontology involves three aspects: biological process (BP), cellular component (CC), and molecular function (MF). The enrichment factor was considered as the cutoff criterion to indicate a statistically significant difference. The top 15 GO terms and KEGG pathways were selected.

### 2.3. Construction and Module Analysis of Protein–Protein Interaction Network

STRING is an online tool designed to evaluate the PPI information. To detect potential relationships among shared DEGs, STRINGapp was used in Cytoscape (version 3.10.4) [18] and mapped the DEGs into STRING [19]. The combined score of >0.4 was used as the cut-off value in the STRING database to improve the result [18]. The Cytoscape app Molecular Complex Detection (MCODE) was applied to create the modules in the PPI network [20], and degree cut-off = 2, node score cut-off = 0.2, k-core = 2, and max depth = 100 were regarded as the criteria. The pathway analysis of genes in each module was performed by DAVID. In addition, GO and KEGG pathway analyses were also conducted to explore the potential information of the genes.

### 2.4. Centrality Analysis of PPI Network

The key genes of the network were identified using the significant parameters of degree centrality, betweenness centrality, closeness centrality, and eigenvector centrality [21]. The four centrality scores of each vertex were calculated by Cytoscape. Degree, betweenness, and closeness were calculated using Network Analyzer of Cytoscape [22], and eigenvector was calculated using Cytoscape app CytoNCA [23]. The score file for these four parameters was downloaded from the Cytoscape software.

### 2.5. The Effect of Expressions of Key Genes on NSCLC Patient Survival

With the widespread use of chips and high-throughput sequencing, a large amount of genomics data has accumulated in the field of cancer research, such as TCGA and ICGC (International Cancer Genome Consortium). Currently, GEPIA (http://gepia.cancer-pku.cn/, accessed on 9 April 2026) is an interactive web server for cancer expression profile data containing 9736 tumor samples and 8587 normal samples from TCGA and GTEx (Genotype-Tissue Expression), which provides customizable functions such as tumor and normal differential expression analysis, and the expression of hub genes in NSCLC tissues and normal ones. *p* < 0.05 was selected as a threshold [24,25].

## 3. Results

### 3.1. Clinical Profiles of the Vietnamese NSCLC Patient Cohort

Table 1 summarizes the clinical characteristics of the 30 patients whose tumor tissues were used for RNA sequencing. Most patients were male (83.3%), had a history of smoking (56.7%), were diagnosed with lung adenocarcinoma (LUAD; 76.7%), and were in advanced stages of disease (IIIb–IV; 76.7%).

### 3.2. Identification of Robust Shared DEGs Across Local and Global Datasets

**Figure 1:**
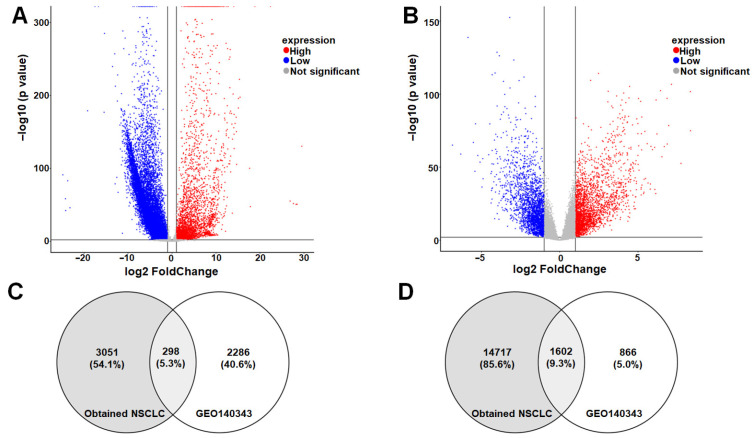
Identification of robust shared DEGs across local and public datasets. (**A**) Volcano plot of the distribution of differentially expressed genes (DEGs) in the Vietnamese NSCLC cohort (Red: High expression; Blue: Low expression). (**B**) Volcano plot of the distribution of DEGs in the GEO140343 dataset. The cut-off criteria were set at |logFC| > 1 and *p*_adj_ < 0.01. (**C**,**D**) Venn diagrams illustrating the intersection of (**C**) high expression and (**D**) low expression genes shared between the local cohort and GSE140343, identifying 1900 shared DEGs.

A total of 19,994 DEGs were identified from the Vietnamese NSCLC cohort and 13,612 DEGs from the GSE140343 dataset. After applying the cut-off criteria (|log2FC| > 1, adjusted *p* < 0.01), 19,668 genes were retained from the Vietnamese dataset, including 3349 high expression genes and 16,319 low expression genes (Figure 1A). In the GSE140343 dataset, 5052 DEGs were identified, comprising 2584 high expression genes and 2468 low expression genes (Figure 1B).

Intersection analysis revealed 298 commonly high expression genes and 1602 commonly low expression genes between the two datasets (Figure 1C,D), resulting in a total of 1900 shared DEGs.

### 3.3. Shared DEGs Are Primarily Enriched in Cancer-Related Pathways and Cell Cycle Processes

**Figure 2:**
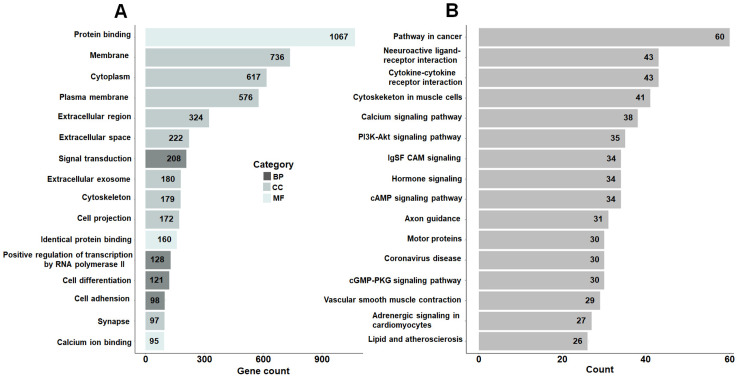
Functional and pathway enrichment of shared DEGs. (**A**) GO functional annotation classifying DEGs into Biological Process (BP), Cellular Component (CC), and Molecular Function (MF). The top 15 terms are ranked by enrichment factor. (**B**) KEGG pathway analysis highlighting the significant involvement of shared DEGs in cancer-related signaling and biological pathways.

To further investigate the biological functions of the shared DEGs, GO, and KEGG enrichment analyses were performed using the DAVID online tool. GO analysis revealed that these genes were significantly enriched in terms related to molecular function, cellular component, and biological process, particularly protein binding, membrane, cytoplasm, plasma membrane, extracellular region, extracellular space, signal transduction, and extracellular exosome (Figure 2A). KEGG pathway analysis indicated that the DEGs were mainly involved in pathways in cancer, neuroactive ligand–receptor interaction, cytokine–cytokine receptor interaction, regulation of actin cytoskeleton, calcium signaling pathway, PI3K–Akt signaling pathway, IgSF cell adhesion molecule signaling, and cAMP signaling pathway (Figure 2B).

### 3.4. Integrated Network Analysis Pinpoints Highly Interconnected Regulatory Hub Genes

**Figure 3:**
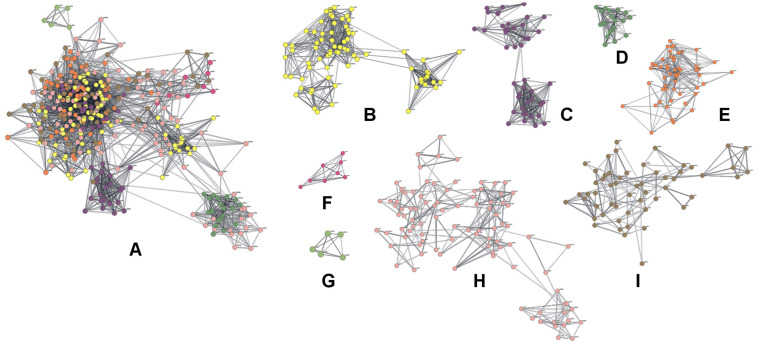
Identification of gene modules within the NSCLC-related PPI network. (**A**) Visualization of the eight top-ranked modules extracted via the MCODE plugin. (**B**–**I**) Detailed structure of Modules 1 through 8. Modules are ranked by MCODE score, representing dense clusters of functionally coordinated genes driving NSCLC pathogenesis.

To identify key regulatory genes driving NSCLC pathogenesis, we mapped 1900 shared DEGs onto the STRING database to construct a PPI network. The resulting network contained 1528 nodes and 8185 edges (Supplementary Figure S1). Lowly expressed genes accounted for the majority of nodes, highlighting their dominant role within the interaction network. While the overarching network was highly dense, applying the MCODE plugin revealed eight highly interconnected modules (Figure 3). These modules represent dense clusters of functionally related genes, serving as a crucial biological bridge between our prior pathway enrichment findings and the subsequent identification of individual hub genes (Supplementary Table S1).

**Figure 4:**
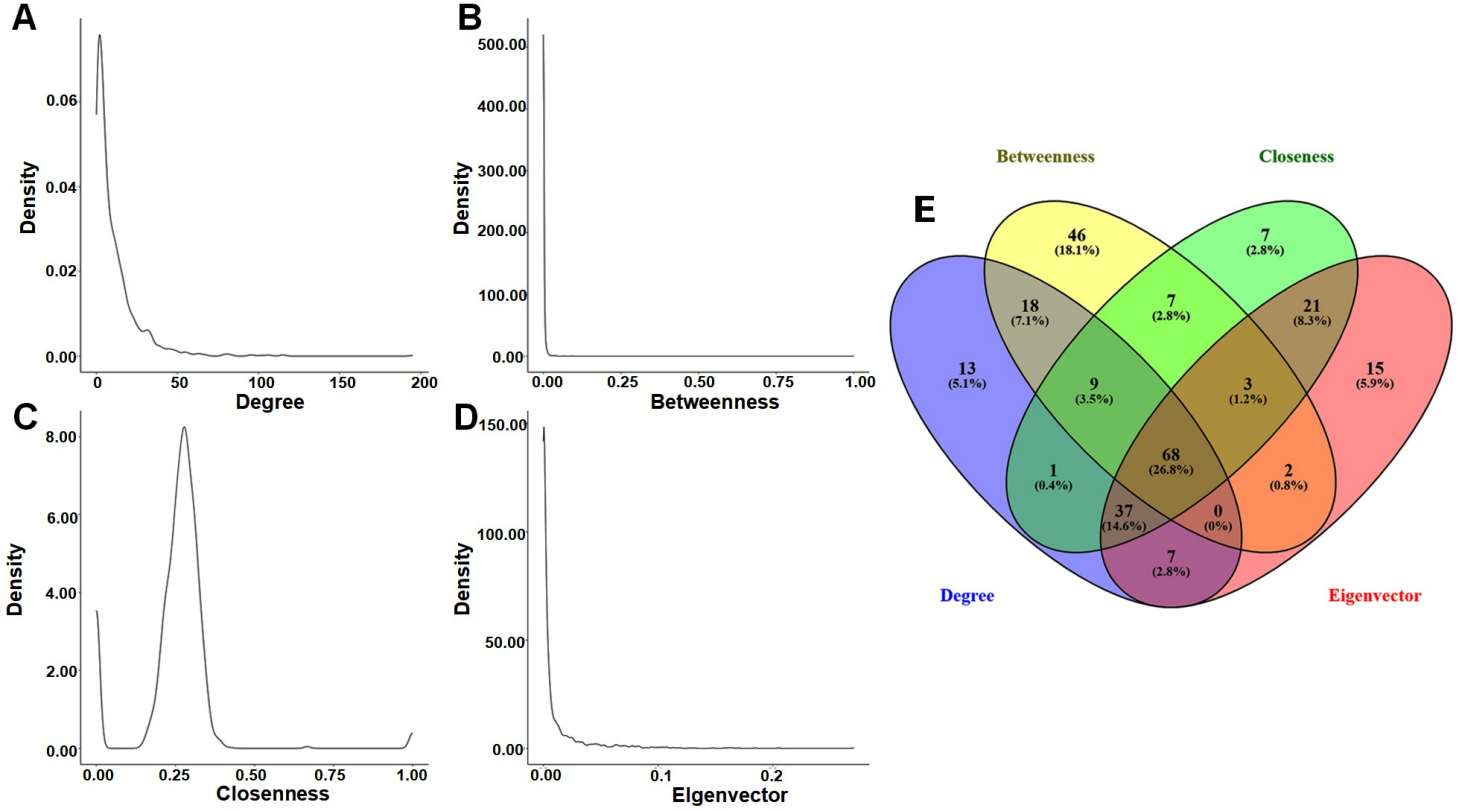
Topological property analysis and hub gene identification. (**A**–**D**) Distribution of degree, betweenness, closeness, and eigenvector centralities, confirming the scale-free and heterogeneous nature of the network. (**E**) Venn diagram illustrating the intersection of the top 10% of nodes across the four centrality measures, pinpointing 68 candidate hub genes with high topological significance.

Beyond these broad structural modules, we performed a centrality analysis to pinpoint the most influential genes within the network. Analysis of the network’s topological properties revealed that the distributions of degree, betweenness, and eigenvector centralities followed power-law patterns, while closeness centrality exhibited a heavy-tailed distribution (Figure 4A–D). These characteristics confirm a scale-free and heterogeneous network structure, typical of robust biological systems. By intersecting the top 10% of nodes across all four centrality measures, we identified 68 key DEGs (Figure 4E). Notably, 64 of these 68 key DEGs were also located within the eight top-ranked modules—including *ADRB2*, *ARRB1*, *CCNA2*, *PTGS2*, *S100A12*, and *SMAD7*—suggesting their central regulatory roles in the NSCLC molecular network (Supplementary Table S2).

### 3.5. Validation of Hub Genes as Potent Prognostic Biomarkers for NSCLC

Due to the lack of long-term follow-up data for the Vietnamese cohort, we evaluated the prognostic significance of the identified hub genes using the GEPIA database, which integrates transcriptomic and clinical data from TCGA and GTEx projects. Patients were stratified into high- and low-expression groups based on median expression levels.

**Figure 5:**
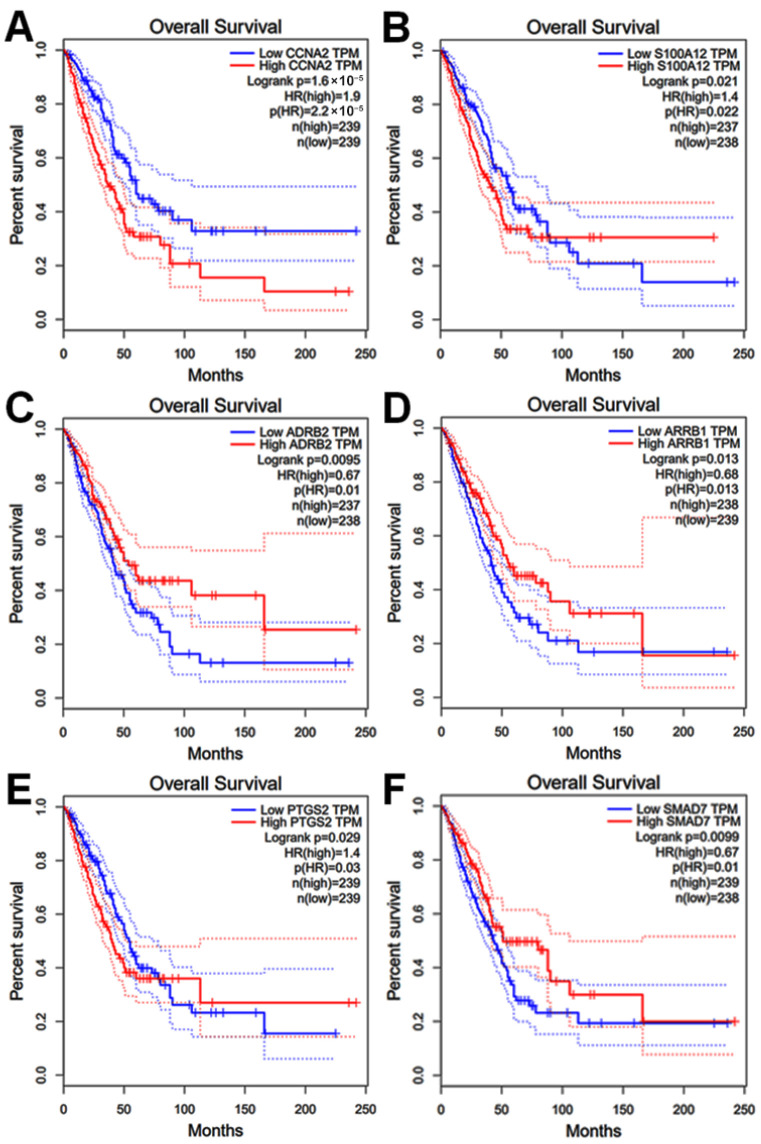
Prognostic value of the identified hub genes in NSCLC. Kaplan–Meier survival curves showing that high expression of (**A**) *CCNA2* and (**B**) *S100A12*, and low expression of (**C**) *ADRB2*, (**D**) *ARRB1*, (**E**) *PTGS2*, and (**F**) *SMAD7* are significantly associated with reduced overall survival (*p* < 0.05). Data were obtained from the GEPIA database, which integrates TCGA and GTEx datasets.

Kaplan–Meier survival analysis revealed that six specific hub genes were significantly associated with patient outcomes (log-rank test, *p* < 0.05; Figure 5A–F). Notably, high expression of *CCNA2* and *S100A12*, as well as low expression of *ADRB2*, *ARRB1*, *PTGS2*, and *SMAD7*, significantly associated with poorer overall survival in NSCLC patients (Figure 5). These results suggest that these six hub genes possess robust prognostic value and may serve as potential prognostic biomarkers.

The consistency between our topological network analysis and clinical survival outcomes reinforces the reliability of these six hub genes. Their significant association with overall survival validates them as core regulatory components of the NSCLC molecular landscape, warranting further functional investigation.

## 4. Discussion

This study integrates transcriptomic data from Vietnamese NSCLC patients with global cohorts to identify a conserved molecular signature across populations. The consistent enrichment of PI3K–Akt, cAMP, and cytokine–receptor signaling highlights their central roles in NSCLC progression. Specifically, the PI3K–Akt pathway acts as a master regulator of protein synthesis and glucose metabolism, providing a survival advantage to tumor cells under the metabolic stress typical of the lung microenvironment [4,5,26]. Network topology and module analyses revealed a scale-free and heavy-tailed architecture with highly interconnected hubs and eight dense functional subnetworks, indicating cooperative molecular modules that drive NSCLC pathogenesis [8,27].

Our analyses highlight six hub genes—*CCNA2*, *S100A12*, *ADRB2*, *ARRB1*, *PTGS2*, and *SMAD7*—as central regulatory nodes with significant prognostic value. *CCNA2* regulates the G1/S and G2/M cell cycle transitions; its high expression in this study is consistent with sustained replicative immortality and the aggressive proliferative phenotype of NSCLC [28,29]. Similarly, *S100A12* drives a pro-tumorigenic microenvironment via RAGE-mediated chronic inflammation and extracellular matrix remodeling, facilitating pre-metastatic niche formation [30,31]. Both genes were associated with poor survival, reinforcing their roles as oncogenic drivers in NSCLC. Conversely, the low expression of ADRB2, ARRB1, PTGS2, and SMAD7 reflects the loss of key regulatory safeguards in NSCLC. Reduced ADRB2 and ARRB1 expression impairs β-adrenergic signaling, weakening control over angiogenesis, cell migration, and immune modulation [32,33]. Likewise, decreased SMAD7, an inhibitory regulator of TGF-β signaling, removes inhibitory control of this pathway, thereby facilitating epithelial–mesenchymal transition (EMT) and enhancing tumor invasiveness [34]. PTGS2 (COX-2) is a well-established mediator of inflammation and tumorigenesis. Although frequently overexpressed in cancer, its low expression in this study was associated with poorer survival, suggesting a context-dependent role. Previous studies have reported that PTGS2 modulation can influence chemoresistance and tumor microenvironment dynamics in NSCLC [35]. Collectively, these alterations highlight how the collapse of inhibitory signaling networks contributes to NSCLC progression and poor patient outcomes.

Notably, this work addresses the underrepresentation of Southeast Asian populations in cancer genomics. By validating our local findings against the well-annotated, predominantly Western TCGA database, we demonstrate that these six biomarkers retain robust prognostic value across diverse genetic backgrounds. This cross-ethnic consistency suggests that these genes are core components of NSCLC biology rather than population-specific artifacts.

Despite these contributions, this study has several limitations. First, the Vietnamese cohort size was modest; however, it was sufficient for exploratory transcriptomic profiling and identifying candidate hub genes when integrated with rigorous validation in large-scale independent datasets. Furthermore, our in silico findings warrant additional functional validation through in vitro and in vivo experiments to fully elucidate the biological mechanisms involved. While future studies incorporating other East Asian cohorts and mechanistic assays will be essential to refine these biomarkers, our work remains a critical first step in expanding precision oncology for underrepresented Southeast Asian populations.

## 5. Conclusions

In summary, module detection and centrality analysis identified six hub genes—*CCNA2*, *S100A12*, *ADRB2*, *ARRB1*, *PTGS2*, and *SMAD7*—as potential key regulators in NSCLC. Survival analysis using TCGA and GTEx data showed that high expression of *CCNA2* and *S100A12*, and low expression of *ADRB2*, *ARRB1*, *PTGS2*, and *SMAD7* were significantly associated with poor overall survival. These findings provide population-relevant molecular insights into NSCLC and suggest that these genes may serve as promising prognostic biomarkers. Further experimental validation and larger, well-annotated clinical cohorts, including diverse Asian populations, are required to clarify their biological functions and clinical utility.

## Figures and Tables

**Table 1:** Demographic and baseline characteristics of NSCLC patients (*n* = 30).

Characteristics	Patients, *n* (%)
Gender	
Male	25 (83.3)
Female	5 (16.7)
Age	
Mean ± SD (range)	65.6 ± 10.7 (41–77)
Median (IQR)	69 (56.5–75)
Smoking habit	
Yes	17 (56.7)
No	13 (43.3)
Histopathologic subtype	
LUAD	23 (76.7)
LUSC	7 (23.3)
Cancer stage	
Early (I–IIIa)	7 (23.3)
Late (IIIb–IV)	23 (76.7)

Data are presented as number (%), mean ± SD, or median (IQR). Abbreviations: NSCLC, non-small-cell lung cancer; LUAD, lung adenocarcinoma; LUSC, lung squamous cell carcinoma. Survival data were not available for the Vietnamese cohort.

## Data Availability

The original contributions presented in this study are included in the article/Supplementary Material. Further inquiries can be directed to the corresponding authors.

## Supplementary Materials

The following supporting information can be downloaded at: https://www.mdpi.com/article/10.3390/cimb48050491/s1.

## Abbreviations

The following abbreviations are used in this manuscript:

NSCLC	Non-small-cell lung cancer
GEO	Gene Expression Omnibus
DEGs	Differentially expressed genes
GO	Gene Ontology
KEGG	Kyoto Encyclopedia of Genes and Genomes
PPI	Protein–protein interaction
TCGA	The Cancer Genome Atlas
ICGC	International Cancer Genome Consortium
LUAD	Lung adenocarcinoma
LUSC	Lung squamous cell carcinoma
RNA-seq	RNA sequencing
cDNA	complementary DNA
