# Supplementary material for: Transcriptomic Profiling Identifies Potential Prognostic Genes in Vietnamese Patients with Non-Small-Cell Lung Cancer
Source: Curr Issues Mol Biol. 2026 May 9;48(5):491. doi: 10.3390/cimb48050491 (PMC13204278; doi:10.3390/cimb48050491)
Supplement: Supplementary file 1 [file cimb-48-00491-s001.zip › cimb-4248946-supplementary.pdf]

SUPPLEMENTARY MATERIALS

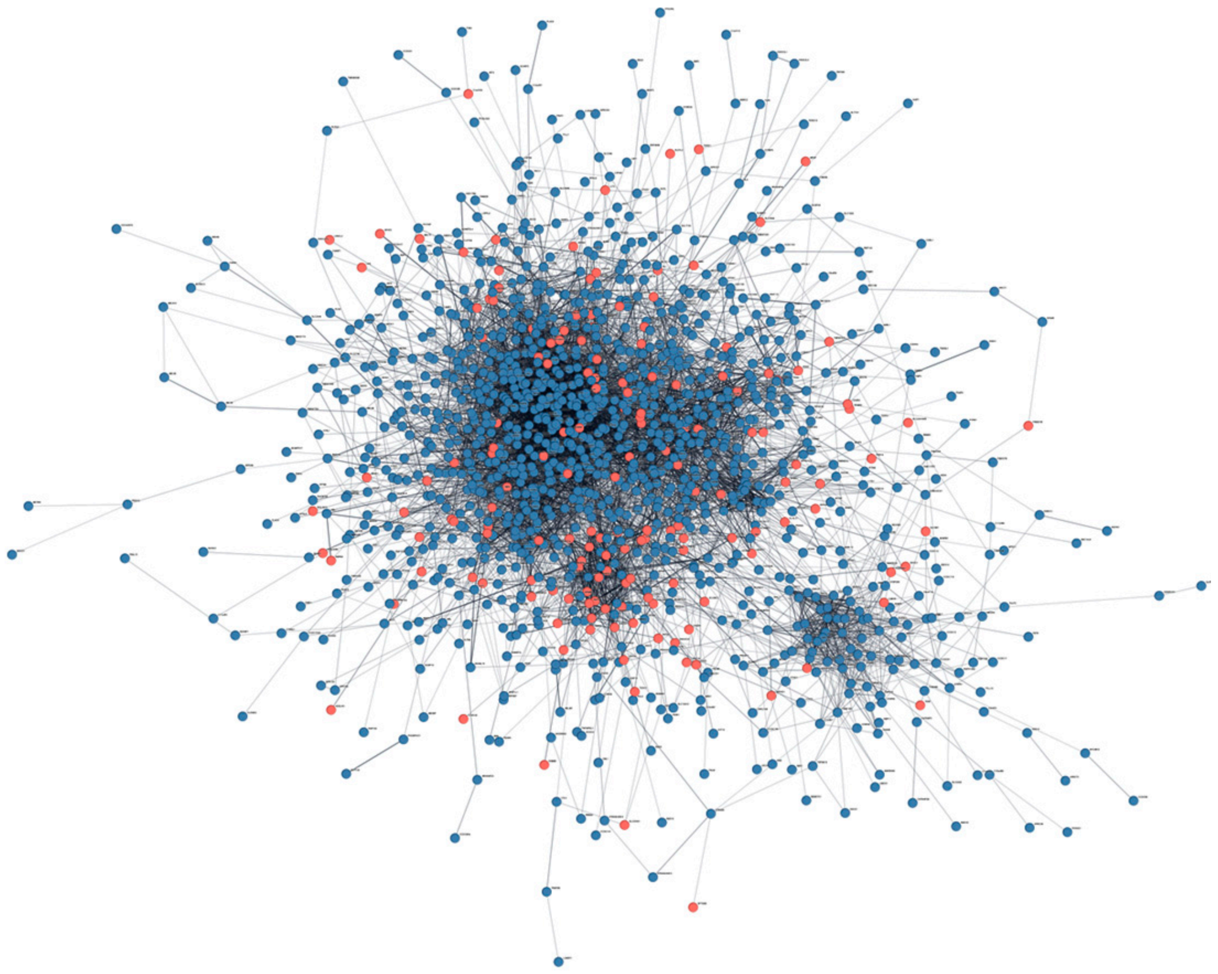

Figure S1. Protein–protein interaction (PPI) network of the 1,900 shared DEGs. The interactome, visualized via the STRING database, comprises 1,528 nodes and 8,185 edges. Red nodes represent high expression genes, while blue nodes represent low expression genes. This network illustrates the complex functional crosstalk and highly interconnected landscape of the identified NSCLC molecular signature.

Table S1. Comprehensive list of nodes, edges, and topological metrics for the eight significant functional sub-networks identified by MCODE analysis.

Module	MCODE score	Number of nodes	Number of edges	Main function
1	13.753	74	502	Angiogenesis and vascular development; inflammatory signaling; Wnt/ MAPK signaling
2	13.636	45	300	Cell cycle regulation; cytokine-mediated inflammatory signaling
3	9.778	19	88	Motile cilium organization; axonemal structure (epithelial function)
4	8.745	52	233	Inflammatory and cytokine signaling; angiogenesis; PPAR-mediated lipid metabolism
5	6.857	8	24	Sensory perception–related processes (primarily auditory genes); potential roles in cell adhesion/receptor activity
6	5	5	11	Metal ion homeostasis; oxidative stress response
7	4.771	97	229	Cytoskeleton organization; cell adhesion; TGF-β signaling (EMT-related)
8	4.37	55	118	Signal transduction (calcium/phosphoinositide); lung epithelial function

Table S2. Detailed topological properties and expression status of 64 candidate hub genes.

Gene	Module	Expression	Degree	Betweenness	Closeness	Eigenvector
A2M	8	low	44	14129.63	0.01	0.05
ACE	1	low	55	22693.48	0.01	0.10
ADRB2	7	low	39	12826.14	0.01	0.04
AGTR1	4	low	62	25444.11	0.01	0.07
AGTR2	8	low	46	10958.01	0.01	0.05
APOA1	4	low	45	14894.23	0.01	0.06
ARG1	1	low	37	10435.08	0.01	0.08
ARRB1	7	low	59	28598.63	0.01	0.05
BMP2	1	low	52	21425.54	0.01	0.07
CAV1	4	low	82	119241.21	0.01	0.10
CCL2	4	low	115	35325.35	0.01	0.20
CCL21	1	low	44	9969.04	0.01	0.07
CCN2	4	low	55	18234.56	0.01	0.10
CCNA2	2	high	38	25380.48	0.01	0.04
CD34	1	low	105	75942.76	0.01	0.16
CD36	1	low	67	34266.76	0.01	0.10
CD69	1	low	40	9255.45	0.01	0.08
CDH5	7	low	80	73138.76	0.01	0.09
CDKN1A	1	low	49	20417.38	0.01	0.09
CEBPA	1	low	50	13303.09	0.01	0.08
CEBPB	2	low	64	12643.29	0.01	0.12
CEBPD	2	low	40	8831.67	0.01	0.08
CLDN5	4	low	48	20874.29	0.01	0.07
CSF3	1	low	63	14897.57	0.01	0.13
DCN	7	low	48	42695.63	0.01	0.05
EDN1	1	low	83	32754.87	0.01	0.13
EDNRB	8	low	29	10473.46	0.01	0.03
EGR1	1	low	63	19335.06	0.01	0.11
EGR2	1	low	42	13275.64	0.01	0.08
ELN	1	low	38	15415.46	0.01	0.06
EPAS1	4	low	35	30092.75	0.01	0.07
ERVFRD-1	7	low	28	11658.49	0.01	0.04
FCGR3B	2	low	67	24240.06	0.01	0.10
FLT4	4	low	33	11082.54	0.01	0.04
FOS	1	low	107	57583.25	0.01	0.16
FOXP3	1	high	56	12364.81	0.01	0.11

Gene	Module	Expression	Degree	Betweenness	Closeness	Eigenvector
GATA1	4	low	40	12481.01	0.01	0.05
GATA2	1	low	50	16718.12	0.01	0.08
GPX3	1	low	35	9086.15	0.01	0.06
IL1A	1	low	79	12363.40	0.01	0.16
IL6	4	low	195	173297.56	0.01	0.27
JUN	1	low	115	65799.93	0.01	0.19
KLF4	2	low	55	9914.02	0.01	0.11
LPL	4	low	36	9483.31	0.01	0.04
MMP1	1	high	33	12851.23	0.01	0.07
NFKBIA	1	low	59	18868.43	0.01	0.12
NOS1	4	low	34	12054.68	0.01	0.05
PDGFB	4	low	45	13542.52	0.01	0.08
PECAM1	4	low	101	48532.50	0.01	0.17
PGR	8	low	32	12733.28	0.01	0.05
PPARG	4	low	96	62963.21	0.01	0.15
PPARGC1A	7	low	50	34006.49	0.01	0.06
PTGS2	1	low	95	45879.87	0.01	0.17
S100A12	4	high	39	9659.91	0.01	0.06
SELE	1	low	54	22380.63	0.01	0.11
SELP	1	low	53	10942.51	0.01	0.09
SMAD7	7	low	31	21676.47	0.01	0.05
SPI1	2	low	56	18105.58	0.01	0.10
TEK	7	low	46	15473.17	0.01	0.07
TGFBR2	4	low	42	15194.15	0.01	0.08
TIE1	1	low	30	13534.48	0.01	0.04
TIMP3	8	low	33	19876.66	0.01	0.05
VWF	1	low	79	52339.68	0.01	0.11
WNT3A	1	low	46	29943.27	0.01	0.05
